# Trophic Condition Shapes UVC Responses in *Euglena gracilis*

**DOI:** 10.3390/life16040539

**Published:** 2026-03-25

**Authors:** Sutthiphat Sriwari, Kittiya Phinyo, Sakawwarin Prommana, Sitthisak Intarasit, Chanenath Sriaporn, Jeeraporn Pekkoh, Sahutchai Inwongwan

**Affiliations:** 1 Department of Biology, Faculty of Science, Chiang Mai University, Chiang Mai 50200, Thailand; sutthiphat_sriwari@cmu.ac.th (S.S.); sakawwarin_pr@cmu.ac.th (S.P.); sitthisak.inta@cmu.ac.th (S.I.); jeeraporn.p@cmu.ac.th (J.P.); 2 Research Group on Space Weather and Cosmic Rays from Ground-Based Observations and Effects on Earth-Space Ecology, Chiang Mai University, Chiang Mai 50200, Thailand; kittiya.ph@cmu.ac.th (K.P.); chanenath.s@cmu.ac.th (C.S.); 3 Algal and Cyanobacterial Research Laboratory, Department of Biology, Faculty of Science, Chiang Mai University, Chiang Mai 50200, Thailand; 4 Office of Research Administration, Chiang Mai University, Chiang Mai 50200, Thailand; 5 Center of Excellence in Microbial Diversity and Sustainable Utilization, Faculty of Science, Chiang Mai University, Chiang Mai 50200, Thailand

**Keywords:** *Euglena gracilis*, ultraviolet C, mixotrophy, oxidative stress, lipid peroxidation

## Abstract

Short-wavelength ultraviolet radiation can impair biological systems by causing DNA damage, oxidative stress, and disruption of photosynthetic processes. Although ultraviolet C (UVC) at 254 nm is widely used as a controlled laboratory stressor, the extent to which trophic condition influences repeated UVC tolerance in phototrophic protists remains unclear. Here, we examined the response of *Euglena gracilis* grown under photoautotrophic or ethanol-supported mixotrophic conditions and exposed to daily UVC pulses for five days. Cell growth, photosynthetic pigments, intracellular oxidative stress measured by 2′,7′ dichlorodihydrofluorescein diacetate fluorescence, and lipid peroxidation estimated as thiobarbituric acid reactive substances equivalent malondialdehyde were assessed, together with qualitative fluorescence microscopy. Repeated UVC exposure reduced cell density in both trophic conditions, with stronger inhibition under photoautotrophy. Photoautotrophic UVC-treated cultures showed the highest oxidative stress signal, whereas malondialdehyde displayed only a non-significant directional increase. Mixotrophic cultures maintained higher cell density under UVC and showed lower oxidative stress signals than photoautotrophic UVC-treated cultures. Pigment responses also differed between trophic conditions, with increased chlorophyll a and carotenoids per cell under photoautotrophic UVC treatment, while mixotrophic pigment levels remained comparatively stable. These findings show that trophic condition shapes repeated UVC stress responses in *E. gracilis* and that ethanol-supported mixotrophy is associated with improved physiological robustness under the present experimental conditions.

## 1. Introduction

Ultraviolet (UV) radiation is an important environmental stressor that can impair cellular function through direct photochemical damage to nucleic acids and proteins, as well as indirect oxidative injury mediated by reactive oxygen species (ROS). In photosynthetic organisms, UV exposure can additionally disrupt photosystem integrity and pigment homeostasis, amplifying redox imbalance under illumination. These effects are relevant not only to terrestrial ecophysiology but also to space biology, where UV radiation contributes to the stress landscape encountered by biological payloads and prospective bioregenerative life support systems (BLSS). In Martian surface scenarios and other low-shielding extraterrestrial contexts, biologically effective UV flux can be elevated relative to present-day Earth due to differences in atmospheric filtering and the absence of an ozone layer, motivating UV stress studies in model organisms [1,2].

Laboratory UVC irradiation (254 nm) provides a widely adopted experimental framework to induce high-energy UV damage in a controlled, reproducible manner. Although UVC is not a direct replica of broadband solar UV spectra, it serves as an informative stressor for testing cellular damage responses, including oxidative injury, pigment remodeling, and survival strategies. In microalgae and phototrophic protists, UVC exposure can trigger growth inhibition, chloroplast disruption, and oxidative stress responses, with outcomes that depend on both exposure regime and intrinsic protective capacity [3]. Importantly, UV sensitivity is not solely determined by dose: biological state variables such as circadian phase, redox buffering capacity, and DNA repair competency can markedly modulate tolerance. For example, *Euglena gracilis* exhibits circadian variation in resistance to UV-C and UV-B exposure, indicating state-dependent regulation of stress defenses beyond simple cell-cycle effects [4]. UV preconditioning can further influence UVC outcomes via photoreactivation-linked processes in *Euglena*, emphasizing the dynamic nature of UV resilience [5].

The unicellular flagellate *E. gracilis* is an especially attractive system for UV stress research because it combines photosynthetic metabolism with robust environmental flexibility and is frequently discussed as a candidate organism for BLSS and space-related biotechnological applications [6]. Its metabolic plasticity allows growth under photoautotrophic, heterotrophic, and mixotrophic regimes, enabling direct evaluation of how carbon and energy supply reshape stress outcomes. Organic carbon availability can profoundly alter cellular physiology by changing ATP generation, reductant balance, and biosynthetic fluxes, which may influence the net oxidative burden experienced under irradiation. Mixotrophic cultivation has been repeatedly reported to enhance biomass productivity and shift metabolic performance in *Euglena* and other microalgal systems [7,8], although the stress-protection consequences of such metabolic states remain less systematically explored. A central challenge in UV stress research is the interpretation of ROS and oxidative stress signal measurements, which are not interchangeable and must be discussed with methodological precision. Consensus guidelines emphasize that commonly used fluorescent probes report composite oxidative signals rather than single ROS species, and that lipid peroxidation assays such as TBARS provide proxy measures that require cautious interpretation and, ideally, complementary validation [9]. Therefore, to rigorously examine trophic-state effects on UVC stress responses in *Euglena*, endpoints should be framed as oxidative stress markers rather than definitive mechanistic proof of specific ROS identity or antioxidant pathway activation.

Despite extensive work on UV-induced stress in photosynthetic microorganisms and the well-documented metabolic plasticity of *E. gracilis*, it remains unclear whether the trophic condition itself alters tolerance to repeated short-wavelength UV challenge. In particular, the extent to which ethanol-supported mixotrophy modifies growth inhibition, pigment remodeling, and oxidative stress markers under repeated UVC exposure has not been systematically examined. Ethanol was selected as the organic carbon source because it is readily assimilated by *E. gracilis* and has been shown to support mixotrophic biomass formation and altered central carbon metabolism in this organism. In this study, we used monochromatic 254 nm UVC as a controlled laboratory stress model rather than an environmental mimic, allowing reproducible comparison of stress responses across trophic conditions. We hypothesized that ethanol-supported mixotrophy would attenuate UVC-associated growth suppression and oxidative stress relative to photoautotrophy by providing greater physiological buffering during repeated irradiation.

## 2. Materials and Methods

### 2.1. Culture Growth and UVC Exposure Experiments

*E. gracilis* CCAP 1224/5Z was maintained in modified Hunter’s medium under sterile conditions. Experimental cultures were inoculated from actively growing exponential phase precultures at 5.0 × 10^4^ cells mL^−1^ into sterile 6-well plates containing 5 mL medium per well. Two trophic conditions were compared: photoautotrophic medium only and ethanol-supported mixotrophic medium containing 1% *v*/*v* ethanol. Ethanol was added only at the start. Cultures were incubated at 25 ± 1 °C under continuous cool white LED illumination at 80 to 100 µmol photons m^−2^ s^−1^. Repeated UVC treatment was carried out in a custom cabinet equipped with low-pressure mercury lamps at 254 nm. Irradiance was measured at the culture exposure plane using a calibrated radiometer and maintained at 2.5 W m^−2^. Plates were positioned at a fixed lamp to culture distance of 100 cm. To minimize attenuation, plate lids were removed during irradiation. Cultures in the UVC treatment were exposed once daily for 100 s for five consecutive days, corresponding to 250 J m^−2^ per day and a cumulative dose of 1.25 kJ m^−2^.

Irradiation was performed in the enclosed UVC cabinet, after which cultures were immediately returned to standard growth conditions. No dedicated dark recovery period was imposed after exposure. Control cultures were handled in parallel but shielded from UVC. Cell density was recorded every 24 h by hemocytometer counting, and cultures were harvested on day 5 for endpoint analyses. For each condition, cells from two wells were pooled to generate one biological replicate, yielding three independent biological replicates per treatment. Pooling was performed to obtain sufficient biomass for endpoint biochemical assays while reducing sampling variation between wells, although this approach may reduce the resolution of within-plate variability.

To contextualize dose selection, the applied UVC regime (254 nm) falls within exposure ranges frequently used in controlled UV stress assays and microbiology irradiation studies, and was used here as a reproducible short-wavelength UV challenge to compare trophic condition-dependent stress responses [10]. The reported dose represents irradiance measured at the exposure plane; minor variation in effective dose may arise from liquid depth and well geometry.

### 2.2. Measurement of Intracellular Reactive Oxygen Species (ROS)

Intracellular ROS levels were quantified using 2′,7′-dichlorodihydrofluorescein diacetate (H_2_DCFDA; Sigma-Aldrich, St. Louis, MO, USA). Aliquots of 300 µL of culture (5.0 × 10^5^ cells mL^−1^) were incubated with 10 µM H_2_DCFDA for 30 min in the dark at 25 °C. Following incubation, fluorescence was measured using a 96-well microplate reader at excitation = 485 nm and emission = 530 nm. Background fluorescence from unstained control samples was subtracted. ROS intensity was normalized to cell number and expressed as relative fluorescence units (RFU) per 10^6^ cells. This normalization facilitates comparison among treatments, but it does not account for possible differences in probe uptake, esterase activity, or intracellular probe oxidation chemistry.

### 2.3. Lipid Peroxidation (MDA Measurement)

Lipid peroxidation was estimated by quantifying malondialdehyde (MDA) using the thiobarbituric acid reactive substances (TBARS) assay (Heath and Packer, 1968 [11]). Approximately 1.0 × 10^6^ cells were harvested by centrifugation (3000× *g*, 10 min), resuspended in 1 mL of 0.1% trichloroacetic acid (TCA), and homogenized on ice. The homogenate (0.5 mL) was mixed with 1.5 mL of 0.5% thiobarbituric acid (TBA) in 20% TCA, heated at 95 °C for 30 min, and then rapidly cooled on ice. After centrifugation (10,000× *g*, 10 min), absorbance was read at 532 nm and 600 nm to correct for nonspecific turbidity. MDA concentration was calculated using an extinction coefficient of 155 mM^−1^ cm^−1^ and expressed as nmol MDA per 10^6^ cells.

### 2.4. Pigment Measurement

For pigment determination, 1 mL of culture containing approximately 5.0 × 10^5^ cells mL^−1^ was centrifuged (3000× *g*, 10 min), and the pellet was extracted with 1 mL of 90% (*v*/*v*) acetone. Samples were vortexed, incubated on ice for 30 min, and centrifuged again to remove debris. Absorbance of the supernatant was recorded at 470 nm, 646 nm, and 663 nm using a microplate spectrophotometer(Molecular Devices, San Jose, CA, USA). Pigments were quantified using the Lichtenthaler and Wellburn equations, which are established for acetone-based spectrophotometric determination of chlorophylls and carotenoids. We selected this solvent system to maintain methodological consistency with the applied equations and to enable direct comparison across treatments [12].

### 2.5. Confocal Microscopy

For structural observations, live and ROS-stained cells were visualized using a confocal laser scanning microscope (Leica Microsystems, Wetzlar, Germany). Aliquots of 300 µL of culture (5.0 × 10^5^ cells mL^−1^) were incubated with 10 µM H_2_DCFDA for 30 min in the dark at 25 °C. Chlorophyll autofluorescence was detected at excitation of 488 nm and emission of 650 to 700 nm. H_2_DCFDA-associated fluorescence was detected at excitation of 488 nm and emission of 510 to 540 nm. Control and UVC-treated samples were imaged under 400× and 1000× magnification. Microscopy was used as qualitative visual support for cellular morphology and fluorescence distribution.

### 2.6. Statistical Analysis

Data are presented as mean ± standard deviation (SD). Endpoint variables, including pigment content, H_2_DCFDA-associated fluorescence, and TBARS-equivalent MDA, were analyzed using two-way ANOVA, with trophic mode and UVC treatment as fixed factors, followed by Tukey’s honestly significant difference post hoc test. Statistical significance was set at *p* < 0.05. Growth data collected over multiple days are shown descriptively across the time course, whereas statistical comparisons reported in the text refer to the day 5 endpoint cell density unless otherwise stated.

## 3. Results

### 3.1. UVC Exposure Inhibits Growth More Severely in Photoautotrophic E. gracilis

**Figure 1:**
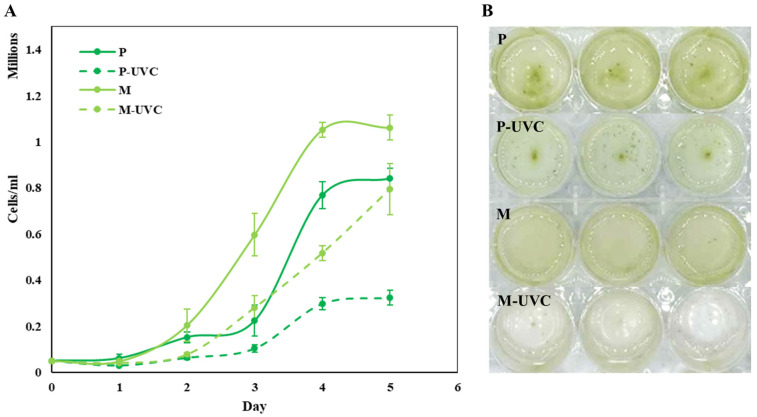
Growth of *E. gracilis* under photoautotrophic and mixotrophic conditions with and without repeated UVC exposure. (**A**) Cultures were grown for 5 days under photoautotrophic (P) or ethanol-supported mixotrophic (M) conditions with or without daily UVC treatment (P-UVC and M-UVC; 254 nm, 2.5 W m^−2^ for 100 s). Cell density (cells mL^−1^) was measured daily. Data are presented as mean ± SD (*n* = 3 biological replicates). (**B**) Representative images of cultures on day 5 under each treatment condition.

Growth kinetics over five days revealed clear trophic-mode differences in both baseline proliferation and sensitivity to repeated UVC exposure (Figure 1). Under control conditions, ethanol-supported mixotrophic cultures (M) achieved a higher final cell density (~1.06 × 10^6^ cells mL^−1^) than photoautotrophic cultures (P; ~0.84 × 10^6^ cells mL^−1^, *p* < 0.05). To evaluate UVC stress responses, cultures were subjected to a daily monochromatic UVC pulse (254 nm, 2.5 W m^−2^ for 100 s; 250 J m^−2^ day^−1^) for five consecutive days (cumulative dose = 1.25 kJ m^−2^). Cell density, quantified daily by hemocytometer counts, showed that repeated UVC exposure reduced population expansion under both trophic modes, but the magnitude of inhibition differed substantially. Photoautotrophic UVC-treated cultures (P-UVC) exhibited the strongest growth suppression and plateaued at ~0.32 × 10^6^ cells mL^−1^ by day 5, whereas mixotrophic UVC-treated cultures (M-UVC) maintained higher densities (~0.80 × 10^6^ cells mL^−1^) relative to their respective controls (Figure 1A). Consistent with these quantitative trends (Figure 1B), endpoint culture appearance indicated reduced visible pigmentation in P-UVC wells compared with M-UVC (Figure 1, inset), suggesting that trophic condition modulates the extent to which repeated UVC stress translates into culture-level biomass and pigmentation outcomes.

### 3.2. Pigment Profiles Shift Under UVC Primarily in Photoautotrophic Cultures

**Figure 2:**
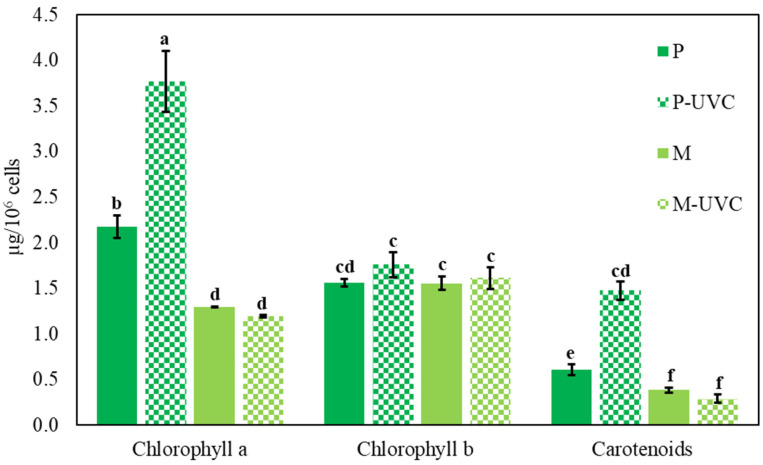
Photosynthetic pigment content of *E. gracilis* under photoautotrophic and mixotrophic conditions with and without repeated UVC exposure. Chlorophyll a, chlorophyll b, and total carotenoids were quantified in photoautotrophic (P) and ethanol-supported mixotrophic (M) cultures with daily UVC treatment (P-UVC and M-UVC) or maintained as non-irradiated controls. Pigment content is expressed as µg × 10^6^ cells^−1^. Data are presented as mean ± SD (*n* = 3 biological replicates). Different letters indicate statistically significant differences among groups (two-way ANOVA with Tukey’s post hoc test, *p* < 0.05).

Photosynthetic pigment quantification revealed a trophic mode-dependent response to repeated UVC exposure (Figure 2). In photoautotrophic cultures, UVC treatment significantly increased chlorophyll a content in P-UVC (3.77 ± 0.33 µg × 10^6^ cells^−1^) compared with the non-irradiated control P (2.18 ± 0.12 µg × 10^6^ cells^−1^, *p* < 0.05). Total carotenoids showed the same trend, increasing from 0.60 ± 0.06 µg × 10^6^ cells^−1^ in P to 1.48 ± 0.10 µg × 10^6^ cells^−1^ in P-UVC (*p* < 0.05). In contrast, mixotrophic cultures displayed no significant UVC-associated changes in chlorophyll a (M: 1.30 ± 0.01 µg × 10^6^ cells^−1^; M-UVC: 1.19 ± 0.02 µg × 10^6^ cells^−1^) or carotenoids (M: 0.38 ± 0.03 µg × 10^6^ cells^−1^; M-UVC: 0.29 ± 0.04 µg × 10^6^ cells^−1^). Chlorophyll b levels remained comparatively stable across all treatments (~1.56–1.76 µg × 10^6^ cells^−1^), with no significant differences detected. Together, these results indicate that repeated UVC exposure is associated with selective increases in chlorophyll a and carotenoids under photoautotrophy, whereas pigment levels remain comparatively stable under mixotrophy, suggesting trophic-condition dependence in pigment remodeling under irradiation stress.

### 3.3. Repeated UVC Exposure Elevates Oxidative Stress Markers, with Stronger Responses Under Photoautotrophy

**Figure 3:**
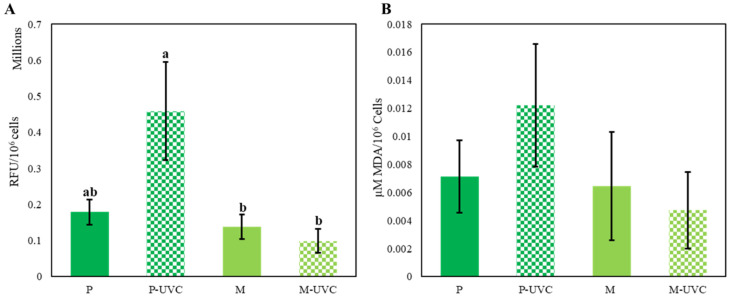
Oxidative stress and lipid peroxidation markers in *E. gracilis* under photoautotrophic and mixotrophic conditions with and without repeated UVC exposure. (**A**) Total oxidative stress signal measured using H_2_DCFDA-derived fluorescence and expressed as arbitrary units (AU) × 10^6^ cells^−1^. (**B**) Lipid peroxidation measured as TBARS-equivalent malondialdehyde (MDA). Photoautotrophic (P) and ethanol-supported mixotrophic (M) cultures were analyzed with or without daily UVC exposure (P-UVC and M-UVC). Data are presented as mean ± SD (*n* = 3 biological replicates). Different letters indicate statistically significant differences among groups (two-way ANOVA with Tukey’s post hoc test, *p* < 0.05). No letters are shown in panel (**B**) because no statistically significant differences were detected among groups.

To evaluate whether repeated UVC exposure altered oxidative stress status, the H_2_DCFDA-derived oxidative stress signal and TBARS-equivalent malondialdehyde (MDA) were quantified at the experimental endpoint (Figure 3A,B). Total oxidative stress signal differed among treatments (Figure 3A), with the highest mean value observed in photoautotrophic UVC-treated cultures (P-UVC; 4.59 × 10^5^ ± 2.35 × 10^5^ AU × 10^6^ cells^−1^, *n* = 3). P-UVC exhibited significantly higher oxidative signal than both mixotrophic groups (M: 1.38 × 10^5^ ± 5.92 × 10^4^ AU × 10^6^ cells^−1^; M-UVC: 9.91 × 10^4^ ± 5.73 × 10^4^ AU × 10^6^ cells^−1^, *p* < 0.05), whereas no significant difference was detected between the two photoautotrophic conditions (P: 1.80 × 10^5^ ± 6.05 × 10^4^ AU × 10^6^ cells^−1^ vs. P-UVC, *p* > 0.05), consistent with the multiple-comparison grouping indicated in Figure 3A.

Lipid peroxidation, measured as TBARS-equivalent MDA, followed a comparable directional pattern, but group differences did not reach statistical significance at *p* < 0.05 (Figure 3B). Overall, these results indicate that repeated UVC exposure is associated with elevated oxidative status primarily in photoautotrophic cultures, while downstream lipid peroxidation showed a consistent trend but remained below the threshold for statistical significance under the current experimental scale.

### 3.4. Fluorescence Imaging Provides Qualitative Support for Trophic Condition-Dependent Differences in Intracellular Fluorescence Patterns

**Figure 4:**
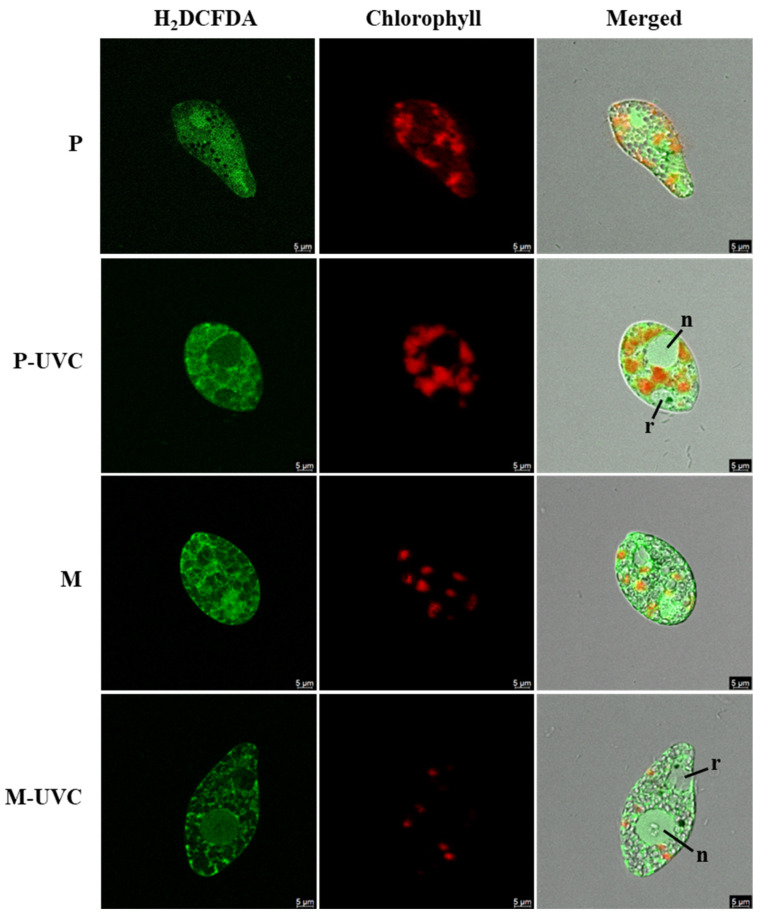
Representative fluorescence microscopy of *E. gracilis* following repeated UVC exposure under photoautotrophic and mixotrophic conditions. Representative images showing H_2_DCFDA-associated oxidative fluorescence (green; (**left column**)), chlorophyll autofluorescence (red; (**middle column**)), and merged fluorescence with bright-field (**right column**) for UVC-treated photoautotrophic (P-UVC) and mixotrophic (M-UVC) cultures. Putative nuclear (n) and reservoir (r) regions are indicated in the merged images. Scale bars = 5 µm.

Fluorescence microscopy provided qualitative visual support for the trophic condition-dependent responses observed in the bulk assays (Figure 4). In UVC-exposed cells, H_2_DCFDA-associated fluorescence in the green channel was observed mainly within the cell interior, consistent with an intracellular oxidative signal. In photoautotrophic UVC-treated cells (P UVC), this signal often appeared visually broader across the cell, whereas in mixotrophic UVC-treated cells (M UVC), it was more frequently restricted to smaller regions.

Chlorophyll autofluorescence in the red channel also showed visible differences between trophic conditions. Mixotrophic cells (M and M UVC) generally displayed less prominent red fluorescence than photoautotrophic cells (P and P UVC), in visual agreement with the pigment trends shown in Figure 2. In merged images, UVC-exposed cells also appeared to show more clearly distinguishable nuclear-like and reservoir-like regions, annotated as n and r. Taken together, these observations are presented as descriptive visual support only and indicate that repeated UVC exposure was accompanied by altered fluorescence patterns that differed between photoautotrophic and mixotrophic states.

## 4. Discussion

### 4.1. Trophic Condition Influences Population Level Tolerance to Repeated UVC Exposure

This study indicates that photoautotrophic *E. gracilis* was more susceptible to repeated UVC exposure than ethanol-supported mixotrophic cultures, as reflected by stronger growth suppression in P-UVC than in M-UVC (Figure 1). This observation aligns with earlier work demonstrating that UV irradiation can cause substantial physiological burden in *Euglena*, including impaired growth and oxidative stress signatures, depending on irradiation intensity and cellular state. For example, oxidative stress and cell damage responses to UV stress have been reported in *E. gracilis* under experimentally controlled exposure conditions, with antioxidant modulation influencing survivability outcomes [3].

The prolonged suppression of population expansion in photoautotrophic UVC-treated cultures may reflect a combination of greater repair burden and reduced metabolic flexibility after repeated irradiation. One plausible explanation is that photoautotrophic cells faced a higher energetic cost for recovery from UVC-induced damage, including repair of nucleic acids and restoration of redox homeostasis. By contrast, ethanol-supported mixotrophy may have provided an additional route for ATP and reductant generation during recovery [7]. This interpretation should also be considered in the context of the cultivation environment, particularly the continuous illumination regime used in the present study. In *E. gracilis*, light conditions can influence chloroplast function, photoreactivation capacity, and the balance between damage and recovery after ultraviolet exposure, indicating that post-irradiation light environment is likely to shape the final phenotype, as well as the trophic state. Prior work has shown that UV-induced lesions in *Euglena* can be repaired through light-dependent photoreactivation, and that the efficiency of this process is affected by the light environment during recovery. Therefore, the stronger suppression observed under photoautotrophy may have reflected not only the absence of an external organic carbon source but also the interaction between trophic mode and the imposed light regime during repeated stress and recovery cycles. Because DNA lesions, repair kinetics, intracellular energy pools, and light-dependent recovery processes were not measured directly in the present study, these interpretations should be regarded as working hypotheses rather than demonstrated mechanisms [5,13,14].

Additionally, *Euglena* exhibits time- and state-dependent UV resistance rhythms, implying that intrinsic physiological context strongly influences UV outcomes beyond dose alone [4]. These results support the view that physiological context modulates UV outcomes in *Euglena*, consistent with earlier reports describing state-dependent sensitivity of *E. gracilis* to UV stress and associated oxidative signatures. Importantly, this growth outcome also aligns with the classic *Euglena* UV literature showing that UV stress can be mitigated by physiological states that favor repair capacity and antioxidant preparedness [15]. *Euglena* exhibits rhythms and inducible responses that modulate UV survival, including circadian variation in UV resistance and photoreactivation of UV-induced DNA lesions [5]. While the present study did not directly measure DNA lesions or repair kinetics, the growth divergence under identical irradiation is consistent with greater physiological resilience under mixotrophy, plausibly by sustaining cellular ATP and reductant supply needed for repair and homeostatic functions.

One interpretation consistent with the growth data is that mixotrophy provides metabolic buffering that may contribute to improved stress tolerance under UVC. *Euglena* is well-known for its metabolic plasticity and ability to shift between phototrophy and organotrophic metabolism depending on environmental inputs, enabling continued energy generation when photosynthetic performance becomes constrained. Studies on *Euglena* and related algae repeatedly show that mixotrophic or organotrophic conditions can elevate biomass productivity and restructure central carbon metabolism and redox balance, often improving physiological robustness under adverse conditions [16,17,18]. In the present study, the higher growth capacity of mixotrophic controls relative to photoautotrophic controls is consistent with the widely recognized advantage of mixotrophy in *Euglena*, where access to an exogenous reduced carbon source supports greater biomass accumulation and metabolic flexibility. Ethanol-driven mixotrophic cultivation has previously been shown to substantially enhance biomass formation and CO_2_ capture compared with photoautotrophy in *E. gracilis*, demonstrating that ethanol can provide a substantial energetic contribution under mixotrophic growth [7]. In this context, the higher cell densities sustained in M-UVC are consistent with a model in which an additional carbon/energy route reduces the probability that UVC-driven cellular damage becomes growth-limiting across the population.

### 4.2. Pigment Remodeling Under UVC Reflects Stress-Associated Acclimation Rather than Demonstrated Photosynthetic Improvement

A notable outcome is that photoautotrophic cultures increased chlorophyll a and carotenoid abundance under UVC, whereas mixotrophic cultures maintained pigment levels comparatively stable. Pigment accumulation in stressed phototrophs does not necessarily indicate improved photosynthetic performance; it may instead reflect changes in cellular allocation, slowed division (leading to higher pigment content per cell), and/or stress-associated pigment remodeling. The stronger response of chlorophyll a compared with chlorophyll b suggests that repeated UVC exposure may have altered pigment allocation in a non-uniform manner. Chlorophyll a is the dominant pigment in reaction center complexes and core photochemical machinery, whereas chlorophyll b is more strongly associated with light-harvesting antenna proteins. The observed increase in chlorophyll a, together with stable chlorophyll b, may therefore reflect selective remodeling of pigment pools during stress acclimation rather than a generalized increase in all photosynthetic pigments. However, in the absence of direct measurements of photochemical performance, this interpretation should remain conservative. In fact, pigment accumulation may coexist with functional impairment if the dominant constraint is photochemical damage, repair bottlenecks, or redox imbalance. In chloroplast-containing systems, ROS production can increase when excitation energy exceeds the capacity for productive electron use, driving oxygen reduction and hydrogen peroxide formation, particularly around PSI/ETC redox bottlenecks [19,20]. Consequently, pigment increases observed in P-UVC should be interpreted as a compensatory phenotype under stress, not as evidence of superior photosynthetic performance.

From a broader astrobiology perspective, pigment-based protection strategies are widely discussed as survival chemistry under harsh irradiation, including UV screening and photoprotectant frameworks in photosynthetic microorganisms [21]. Although scytonemin-based strategies are best characterized in cyanobacteria rather than *Euglena*, the conceptual link supports why photoautotrophic cells may display stronger pigment remodeling under UVC as part of a stress-response signature rather than a productivity gain. One defensible interpretation is that P-UVC pigment increases reflect a stress-acclimation response in which cells attempt to maintain effective light harvesting and photoprotection during damage pressure. Carotenoids play central roles in photoprotection by dissipating excess excitation energy and quenching reactive intermediates (including singlet oxygen), thereby protecting photosystems from oxidative injury [22]. Algal carotenoid composition and photoprotective function are highly diverse, and carotenoids are widely recognized as key oxidative stress buffers in oxygenic phototrophs [23]. Therefore, a UVC-driven carotenoid rise in photoautotrophic conditions is consistent with selective pressure for photoprotective capacity when photosynthetic electron transport is challenged.

In contrast, pigment profiles in mixotrophic cultures remained comparatively stable and lower on a per-cell basis. This may reflect that mixotrophy shifts cellular energy/redox management and reduces the need for major pigment rebalancing under the same irradiation regime. This interpretation is supported by comparative proteomics and metabolomics evidence showing that central carbon metabolism and broader cellular physiology differ substantially between phototrophic, mixotrophic, and heterotrophic states in *E. gracilis* [24,25]. In closed culture systems, *Euglena* physiology is known to depend on light regimes, and changes in light availability can influence photosynthesis-linked behavior and performance [26]. These established dependencies support the interpretation that trophic and light context together shape pigment phenotypes and stress outcomes.

In contrast, pigment stability in mixotrophy likely reflects reduced reliance on photosynthesis as the sole energy generator, which can reduce excitation pressure and lower the tendency toward ROS amplification at the photosystems. This interpretation is consistent with the broader understanding that metabolic flexibility can relieve redox congestion and redistribute electron flow away from oxygen reduction pathways when photosynthetic metabolism is destabilized [27,28]. Taken together, the pigment data support a model in which photoautotrophic cells respond to UVC by adjusting pigment pools (including carotenoids), whereas mixotrophic cells maintain pigment homeostasis because their growth and survival are not as tightly coupled to photosynthetic throughput. Although chlorophyll a and carotenoid abundance increased in photoautotrophic UVC-treated cultures, these shifts should not be interpreted as evidence of improved photosynthetic function. Pigment accumulation can accompany stress-associated remodeling, reduced cell division, or compensatory reallocation, and direct measurements such as Fv/Fm, non-photochemical quenching, and electron transport-related parameters would be required to establish functional consequences.

### 4.3. Oxidative Stress Markers Indicate Stronger Photoautotrophic Stress Burden but Not Definitive Oxidative Damage

The oxidative stress assays show a coherent pattern: the highest H_2_DCFDA-derived oxidative signal occurs in photoautotrophic UVC-treated cultures (P-UVC), which is significantly elevated relative to both mixotrophic groups (M and M-UVC), while P and P-UVC are not statistically separable due to high variability. This broadly supports the conclusion that UVC exposure under photoautotrophy imposes a larger oxidative burden than under mixotrophy, consistent with earlier demonstrations of oxidative stress-mediated UV injury in *E. gracilis*. UVC triggers cellular stress through both direct photochemical damage and indirect ROS generation [3,15]. In *Euglena*, oxidative stress responses to UV and related ROS-inducing conditions are well-documented, and antioxidant supplementation can suppress UV-induced injury. The present study extends this framework by demonstrating that metabolic state alone, without external antioxidant supplementation, modulates the oxidative outcome under UVC.

At the same time, interpretation of the H_2_DCFDA signal should remain cautious because normalization to cell number does not eliminate potential variability arising from dye uptake, intracellular esterase activity, or differential probe oxidation among trophic conditions. H_2_DCFDA-based readouts are widely used to track oxidative stress, but they do not resolve ROS species, can be influenced by cellular uptake/esterase activity, and may reflect a composite oxidative environment rather than a single oxidant. Future work should therefore combine this assay with orthogonal endpoints such as superoxide-specific detection, hydrogen peroxide-focused assays, and antioxidant enzyme activities, including superoxide dismutase, catalase, and peroxidase-related measurements. Current best-practice consensus guidelines emphasize that ROS assays require cautious interpretation and ideally triangulation across orthogonal methods and damage markers [9,29]. Likewise, TBARS-based MDA quantification is a useful but non-specific marker, as the assay can react with multiple aldehydic by-products and is sensitive to sample handling and chemistry [30]. This is particularly relevant here because lipid peroxidation (TBARS-equivalent MDA) follows a similar directional trend but does not reach statistical significance. TBARS assays are known to be chemically non-specific and may overestimate or obscure true MDA differences depending on interfering compounds, sample matrix, and reaction conditions; thus, this may reflect biological reality, limited sample size, or assay sensitivity constraints [31].

From a systems perspective, P-UVC likely experiences ROS amplification from both photosynthetic and mitochondrial contributions. Photosynthetic electron transport can generate superoxide and hydrogen peroxide when electron acceptor capacity is limited and oxygen becomes a competing sink [19,20]. In parallel, mitochondrial respiration can become a major ROS driver under stress when the electron transport chain redox balance is disturbed. In photoautotrophy, the cell’s energy economy is tightly dependent on light-driven processes; thus, UVC-driven disruption can produce a combined burden: damage to photochemistry, increased ROS, and greater lipid peroxidation, which together suppress growth. Mixotrophy plausibly buffers these cascades by sustaining ATP and NAD(P)H availability through carbon assimilation routes that do not require fully intact photosystem function. This buffering can reduce the probability of ROS runaway because antioxidant defense (e.g., glutathione/ascorbate systems and peroxidases) and repair processes are energy- and reductant-dependent themselves. This interpretation fits the broader logic of redox regulation and acclimation in photosynthetic organisms, where ROS production reflects not only stress intensity but also the cell’s capacity to dissipate excitation energy and maintain reductive homeostasis [32].

Fluorescence imaging showed condition-dependent intracellular patterns that qualitatively agree with the bulk measurements. Under repeated UVC exposure, the H_2_DCFDA-associated signal appeared visually broader in photoautotrophic cells than in mixotrophic cells, consistent with a stronger oxidative stress signature in P-UVC relative to M-UVC. Likewise, the generally lower chlorophyll autofluorescence intensity under mixotrophy compared with photoautotrophy aligns with the per-cell pigment profiles. In the merged views, UVC-exposed cells frequently exhibited more prominent nuclear- and reservoir-like regions (n and r); while these features provide useful morphological descriptors, their functional interpretation should remain conservative without dedicated structural markers. This caution is warranted because *Euglena* photobehavior and cell physiology can be influenced by UV exposure, affecting motility and phototaxis and reflecting broader shifts in cellular organization [33]. Moreover, the reservoir in *E. gracilis* is a defined anterior invagination associated with the flagellar apparatus and has been characterized ultrastructurally by SEM [34], supporting the anatomical plausibility of the annotated region in our images. Finally, fluorescence-based imaging under deep-UV/near-UV excitation has been advanced in astrobiology-oriented instrumentation as a sensitive approach for detecting microbial signatures and stress-associated phenotypes [35]. However, the microscopy data are descriptive and should be interpreted as visual support for the bulk measurements rather than as statistically validated evidence of subcellular reorganization.

## 5. Conclusions

Trophic condition significantly shaped the response of *E. gracilis* to repeated UVC exposure. Under the same irradiation regime, ethanol-supported mixotrophy maintained higher cell density and a lower H_2_DCFDA-derived oxidative stress signal than photoautotrophy. In contrast, pigment remodeling was more pronounced in photoautotrophic cultures, while TBARS-equivalent MDA showed only a non-significant directional trend. Overall, these findings indicate that mixotrophic cultivation can improve physiological robustness under repeated UVC stress, while the underlying mechanisms remain to be resolved through direct analysis of photosynthetic performance, antioxidant systems, and DNA damage or repair pathways. Although *E. gracilis* is of interest in controlled environment and space-related bioprocess research, the present experiment was conducted under Earth laboratory conditions using monochromatic 254 nm UVC and should not be interpreted as a direct simulation of planetary UV environments.

## Data Availability

The original contributions presented in this study are included in the article. Further inquiries can be directed to the corresponding author.

## References

[1] Cockell C.S., Andrady A.L. (1999). The Martian and extraterrestrial UV radiation environment—1. Biological and closed-loop ecosystem considerations. Acta Astronauti..

[2] Cockell C.S., Catling D.C., Davis W.L., Snook K., Kepner R.L., Lee P., McKay C.P. (2000). The Ultraviolet Environment of Mars: Biological Implications Past, Present, and Future. Icarus.

[3] Palmer H., Ohta M., Watanabe M., Suzuki T. (2002). Oxidative stress-induced cellular damage caused by UV and methyl viologen in Euglena gracilis and its suppression with rutin. J. Photochem. Photobiol. B.

[4] Bolige A., Kiyota M., Goto K. (2005). Circadian rhythms of resistance to UV-C and UV-B radiation in Euglena as related to ‘escape from light’ and ‘resistance to light’. J. Photochem. Photobiol. B.

[5] Takahashi A., Shibata N., Nishikawa S., Ohnishi K., Ishioka N., Ohnishi T. (2006). UV-B light induces an adaptive response to UV-C exposure via photoreactivation activity in Euglena gracilis. Photochem. Photobiol. Sci..

[6] Häder D.P. (2020). On the Way to Mars—Flagellated Algae in Bioregenerative Life Support Systems Under Microgravity Conditions. Front. Plant Sci..

[7] Inwongwan S., Duangjan K., Sensupa P., Phinyo K., Ruangrit K., Pumas C., Pekkoh J. (2025). Ethanol-driven mixotrophic cultivation enhances biomass production and CO_2_ capture in Euglena gracilis for sustainable bioresource utilisation. Algal Res..

[8] Toyama T., Hanaoka T., Yamada K., Suzuki K., Tanaka Y., Morikawa M., Mori K. (2019). Enhanced production of biomass and lipids by Euglena gracilis via co-culturing with a microalga growth-promoting bacterium, Emticicia sp. EG3. Biotechnol. Biofuels.

[9] Murphy M.P., Bayir H., Belousov V., Chang C.J., Davies K.J.A., Davies M.J., Dick T.P., Finkel T., Forman H.J., Janssen-Heininger Y. (2022). Guidelines for measuring reactive oxygen species and oxidative damage in cells and in vivo. Nat. Metab..

[10] Yemmireddy V., Adhikari A., Moreira J. (2022). Effect of ultraviolet light treatment on microbiological safety and quality of fresh produce: An overview. Front. Nutr..

[11] Heath R.L., Packer L. (1968). Photoperoxidation in isolated chloroplasts: I. Kinetics and stoichiometry of fatty acid peroxidation. Arch. Biochem. Biophys..

[12] Lichtenthaler H.K., Wellburn A.R. (1983). Determinations of total carotenoids and chlorophylls a and b of leaf extracts in different solvents. Biochem. Soc. Trans..

[13] Srinivas U., Lyman H. (1980). Photomorphogenic Regulation of Chloroplast Replication in Euglena: ENHANCED LOSS OF CHLOROPLAST DNA IN RED LIGHT. Plant Physiol..

[14] Nicolas P., Hussein Y., Heizmann P., Nigon V. (1980). Comparative studies of chloroplastic and nuclear DNA repair abilities after ultraviolet irradiation of Euglena gracilis. Mol. Genet. Genom..

[15] Bagnato C., Nadal M.S., Tobia D., Raineri M., Vasquez Mansilla M., Winkler E.L., Zysler R.D., Lima E. (2021). Reactive Oxygen Species in Emulated Martian Conditions and Their Effect on the Viability of the Unicellular Alga Scenedesmus dimorphus. Astrobiology.

[16] Shan S., Manyakhin A.Y., Wang C., Ge B., Han J., Zhang X., Zhou C., Yan X., Ruan R., Cheng P. (2023). Mixotrophy, a more promising culture mode: Multi-faceted elaboration of carbon and energy metabolism mechanisms to optimize microalgae culture. Bioresour. Technol..

[17] Yamane Y.-i., Utsunomiya T., Watanabe M., Sasaki K. (2001). Biomass production in mixotrophic culture of *Euglena gracilis* under acidic condition and its growth energetics. Biotechnol. Lett..

[18] Nakazawa M. (2017). C2 metabolism in Euglena. Adv. Exp. Med. Biol..

[19] Asada K. (2006). Production and Scavenging of Reactive Oxygen Species in Chloroplasts and Their Functions. Plant Physiol..

[20] Kozuleva M.A., Ivanov B.N., Vetoshkina D.V., Borisova-Mubarakshina M.M. (2020). Minimizing an Electron Flow to Molecular Oxygen in Photosynthetic Electron Transfer Chain: An Evolutionary View. Front. Plant Sci..

[21] Varnali T., Edwards H.G. (2010). Iron-scytonemin complexes: DFT calculations on new UV protectants for terrestrial cyanobacteria and astrobiological implications. Astrobiology.

[22] Ramel F., Birtic S., Cuiné S., Triantaphylidès C., Ravanat J.L., Havaux M. (2012). Chemical quenching of singlet oxygen by carotenoids in plants. Plant Physiol..

[23] Ramel F., Mialoundama A.S., Havaux M. (2012). Nonenzymic carotenoid oxidation and photooxidative stress signalling in plants. J. Exp. Bot..

[24] Hasan M.T., Sun A., Khatiwada B., McQuade L., Mirzaei M., Te’o J., Hobba G., Sunna A., Nevalainen H. (2019). Comparative proteomics investigation of central carbon metabolism in Euglena gracilis grown under predominantly phototrophic, mixotrophic and heterotrophic cultivations. Algal Res..

[25] Inwongwan S., Sriwari S., Pumas C. (2025). Metabolomic Insights into the Adaptations and Biotechnological Potential of Euglena gracilis Under Different Trophic Conditions. Plants.

[26] Richter P.R., Strauch S.M., Ntefidou M., Schuster M., Daiker V., Nasir A., Haag F.W., Lebert M. (2014). Influence of different light-dark cycles on motility and photosynthesis of Euglena gracilis in closed bioreactors. Astrobiology.

[27] Saint-Sorny M., Brzezowski P., Arrivault S., Alric J., Johnson X. (2022). Interactions between carbon metabolism and photosynthetic electron transport in a *Chlamydomonas reinhardtii* mutant without CO_2_ fixation by RuBisCO. Front. Plant Sci..

[28] Walker B.J., Kramer D.M., Fisher N., Fu X. (2020). Flexibility in the Energy Balancing Network of Photosynthesis Enables Safe Operation under Changing Environmental Conditions. Plants.

[29] Akter S., Khan M.S., Smith E.N., Flashman E. (2021). Measuring ROS and redox markers in plant cells. RSC Chem. Biol..

[30] Aguilar Diaz De Leon J., Borges C.R. (2020). Evaluation of Oxidative Stress in Biological Samples Using the Thiobarbituric Acid Reactive Substances Assay. J. Vis. Exp..

[31] Moselhy H.F., Reid R.G., Yousef S., Boyle S.P. (2013). A specific, accurate, and sensitive measure of total plasma malondialdehyde by HPLC. J. Lipid Res..

[32] Foyer C.H., Noctor G. (2005). Redox homeostasis and antioxidant signaling: A metabolic interface between stress perception and physiological responses. Plant Cell.

[33] Hauder D.-P. (1986). Effects of solar and artificial uv irradiation on motility and phototaxis in the flagellate, *Euglena gracilis*. Photochem. Photobiol..

[34] Gualtieri P., Barsanti L., Passarelli V., Verni F., Rosati G. (1990). A look into the reservoir of euglena gracilis: SEM investigations of the flagellar apparatus. Micron Microsc. Acta.

[35] Case N., Johnston N., Nadeau J. (2024). Fluorescence Microscopy with Deep UV, Near UV, and Visible Excitation for In Situ Detection of Microorganisms. Astrobiology.

